# Trend and early clinical outcomes of off-pump coronary artery bypass grafting in the UK

**DOI:** 10.1093/ejcts/ezad272

**Published:** 2023-07-31

**Authors:** Jeremy Chan, Arnaldo Dimagli, Tim Dong, Daniel P Fudulu, Shubhra Sinha, Gianni D Angelini

**Affiliations:** Bristol Heart Institute, University of Bristol, Bristol, UK; Bristol Heart Institute, University of Bristol, Bristol, UK; Bristol Heart Institute, University of Bristol, Bristol, UK; Bristol Heart Institute, University of Bristol, Bristol, UK; Bristol Heart Institute, University of Bristol, Bristol, UK; Bristol Heart Institute, University of Bristol, Bristol, UK

**Keywords:** Cardiopulmonary bypass, Off-pump coronary artery bypass grafting

## Abstract

**OBJECTIVES:**

The popularity of off-pump coronary artery bypass grafting (CABG) varies across the world, ranging from 20% in Europe and the USA to 56% in Asia. We present the trend and early clinical outcomes in off pump in the UK.

**METHODS:**

All patients who underwent elective or urgent isolated CABG from 1996 to 2019 were extracted from the National Adult Cardiac Surgery Audit database. The trend in operating surgeons and units volume and training in off pump were analysed. Early clinical outcomes between off- and on-pump CABG were compared using propensity score matching.

**RESULTS:**

A total of 351 422 patients were included. The overall off-pump rate during the study period was 15.17%, it peaked in 2008 (19.8%), followed by a steady decreased to 2018 (7.63%). Its adoption varied across centres and surgeons, ranging from <1% to 48.36% and <1% to 85.5%, respectively, of total cases performed. After propensity score matching for the period 1996–2019, off pump, when compared to on pump, was associated with a lower in-hospital/30-day mortality (1.2% vs 1.5%, *P* < 0.001), return to theatre (3.7% vs 4.5%, *P* < 0.001), cerebrovascular accident (transient ischaemic attack: 0.3% vs 0.6%, stroke: 0.3% vs 0.6%, *P* < 0.001) and deep sternal wound infection (0.8% vs 1.2%, *P* ≤ 0.001). In a sub-analysis from the introduction of EuroScore II (2012–2019), there were no differences in-hospital/30-day mortality (1.0% vs 1.0%, *P* = 0.71). However, on pump, had a higher return to theatre (4.2% vs 2.7%, *P* < 0.001), cerebrovascular accident (transient ischaemic attack: 0.4% vs 0.2%, stroke: 0.5% vs 0.3%, *P* = 0.003) and deep sternal wound infection (1.0% vs 0.6%, *P* = 0.004).

**CONCLUSIONS:**

Our data show a decreasing trend in the use of off pump in the UK since 2008. This is likely to be multifactorial and raises the question of whether it should be a specialized revascularization technique.

**Figure:**
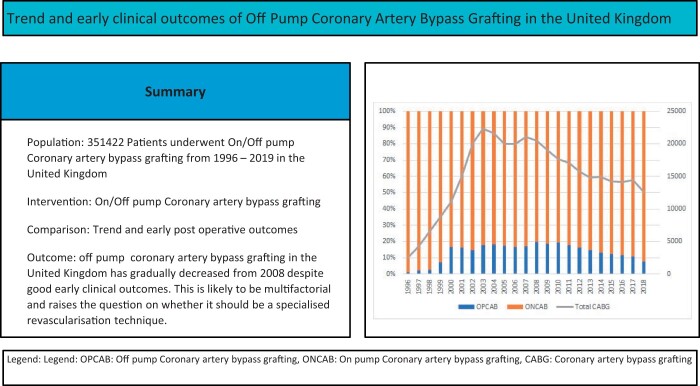

## INTRODUCTION

Coronary artery bypass grafting (CABG) is traditionally performed with cardiopulmonary bypass, allowing surgeons to operate on a bloodless field with an arrested heart. Off-pump coronary artery bypass (OPCAB) was first reported in the 1960s, with the operation performed on a beating heart [1]. It has been suggested that by avoiding the inflammatory response from cardiopulmonary bypass, patients, particularly in certain high risks groups like octogenarians, those with pulmonary disease, poor left ventricular functions, high EuroScore II and porcelain aorta, could most benefit from OPCAB [2].

However, several randomized control trials (RCTs) and many retrospective cohort studies have not shown any conclusive major differences in short- and long-term mortality between the 2 revascularization techniques resulting in different popularity of OPCAB across the world, ranging from 20–25% in Europe and the USA to 50–70% in Asia [3, 4]. There are limited studies reporting the trend in OPCAB in recent years in the literature [5, 6]. We, therefore, aimed to present the trend and the early clinical outcomes in OPCAB in the UK over a 23-year period. Furthermore, using propensity score matching (PSM), we investigated differences in early clinical outcome between OPCAB and on-pump coronary artery bypass (ONCAB).

## METHODS

All patients who underwent elective or urgent isolated CABG from April 1996 to April 2019 (in the UK data are collected according to the financial year April to April) were extracted from the National Adult Cardiac Surgery Audit (NACSA) database. The NACSA database prospectively collects data on all major heart operations carried out on National Health Service patients in the UK since April 1996. The definitions of database variables used, and a description of the database was previously described [7]. Patients were divided into 2 groups: (i) ONCAB and (ii) OPCAB. Patients who underwent emergency or salvage CABG, non-isolated CABG and had previous cardiac surgery (re-do cases) were excluded from the study. Multiple arterial grafting was defined as ≥2 arterial grafts used. Patients with missing data regarding the use of cardiopulmonary bypass (*n* = 9384, 2.69%) were also excluded from the analysis. The trend, early clinical outcomes, individual surgeon and unit volume and trainees' exposure to OPCAB technique were compared. The primary outcome was in-hospital/30-day mortality. Furthermore, using PSM, we compared the early clinical outcomes between the 2 revascularization techniques. Furthermore, we compared the short-term postoperative outcomes between the 2 revascularization techniques in certain high-risk groups—patients with preoperative pulmonary disease (chronic pulmonary disease requiring the use of long-term medication), poor left ventricular ejection fraction (<30%), extracardiac arteriopathy and EuroScore II≥4. Primary/first operator should performed >= 50% of the total number of anastomosis. Trainee was defined as non-consultant surgeons, regardless of experience.

The observed mortality was compared against the expected mortality in all patients, and ONCAB and OPCAB groups. The observed mortality was defined as 30-day or in-hospital mortality after the index operation. The expected mortality was calculated using the EuroScore II.

### Ethical statement

The study was part of a research project approved by the Health Research Authority and Health and Care Research Wales. As the study included retrospective interrogation of the NICOR database the need for individual patient consent was waived off (Health and Care Research Wales) (IRAS ID: 278171) in accordance with the research guidance. The study was performed in accordance with the ethical standards as laid down in the 1964 Declaration of Helsinki and its later amendments.

### Statistical analysis

Continuous variables are presented as mean [standard deviation (SD)] or median (interquartile range) and were compared using Student’s *t*-test or Wilcoxon rank-sum test, as appropriate. Categorical variables are presented as numbers and frequencies and were compared using chi-squared exact test. Missing continuous variables data (body mass index, *n* = 23 527, left ventricular ejection fraction category, *n* = 6585) were imputed with the median value in the data after the application of exclusion criteria listed above, while categorical variable (gender *n* = 207) was imputed with the mode.

To account for potential imbalance in baseline risk, a propensity score was calculated for each patient based on a non-parsimonious logistic regression model. All variables included in the model are listed in Table 1. A 1:1 nearest-neighbour PSM without replacement and with a calliper width of 0.2 SDs of the logit of the propensity score was performed. After PSM, standardized mean difference was used to assess balance of the covariates between the ONCAB and OPCAB groups. A value higher than 0.10 was considered to indicate the presence of residual imbalance among variables. The covariate balance before and after as well as the effectiveness of the PSM was visualized (Supplementary Material, Figs S1 and S2). A generalized, linear model was used to evaluate the factors predicting the adoption of OPCAB. Results are demonstrated as odds ratio (OR) and 95% confidence interval (CI). In all the analyses, ONCAB was used as the reference group. R (Version 4.2.0) and R Studio (Version 1.4.1103, RStudio, PBC) were used to perform the statistical analysis. A *P*-value of <0.05 is deemed statistically significant.

**Table 1:** The preoperative characteristics between off-pump coronary artery bypass graft and on-pump coronary artery bypass before and after propensity score matching (1996–2019)

Preoperative characteristics	Pre-PSM			Post-PSM			
	ONCAB (*n* = 298 119)	OPCAB (*n* = 53 303)	*P*-Value	ONCAB (*n* = 53 301)	OPCAB (*n* = 53 301)	SMD	*P*-Value
Age (years), mean (SD)	65.87 (9.50)	65.81 (9.94)	>0.99	65.81 (9.94)	65.83 (9.48)	0.0996	0.49
Gender, *n* (%)			<0.001				0.029
Male	242 494 (81)	42 821 (80)		42 820 (80)	43 102 (81)	0.0017	
Female	55 625 (19)	10 482 (20)		10 481 (20)	10 199 (19)	0.0136	
BMI, mean (SD)	28.48 (4.62)	28.33 (4.68)	<0.001	28.33 (4.68)	28.46 (4.47)	–0.0136	<0.001
LVEFC, *n* (%)			<0.001				
Very poor (LVEF <21%)	108 (<0.1)	3 (<0.1)		3 (<0.1)	5 (<0.1)	0.0276	
Poor (LVEF 21–30%)	84 593 (28)	13 688 (26)		13 688 (26)	14 917 (28)	0.002	
Moderate (LVEF 31–50%)	4286 (1.4)	341 (0.6)		341 (0.6)	395 (0.7)	0.0511	
Good (LVEF >50%)	209 132 (70)	39 271 (74)		39 269 (74)	37 984 (71)	0.0085	
Urgency, *n* (%)			<0.001				<0.001
Elective	195 731 (66)	36 156 (68)		36 156 (68)	35 350 (66)	–0.0527	
Urgent	102 388 (34)	17 147 (32)		17 145 (32)	17 951 (34)	–0.0318	
Diabetes, *n* (%)			<0.001				0.001
Not diabetic	222 462 (75)	40 625 (76)		40 624 (76)	40 073 (75)	0.0318	
Diet control	12 310 (4.1)	2056 (3.9)		2056 (3.9)	2156 (4.0)	–0.0238	
Oral therapy	40 918 (14)	6728 (13)		6728 (13)	7054 (13)	0.0094	
Insulin therapy	22 429 (7.5)	3894 (7.3)		3893 (7.3)	4018 (7.5)	0.0178	
Smoking, *n* (%)			<0.001				0.069
Never smoked	97 670 (33)	17 477 (33)		17 476 (33)	17 444 (33)	0.0089	
Ex-smoker	165 888 (56)	29 356 (55)		29 356 (55)	29 618 (56)	–0.0013	
Current smoker	34 561 (12)	6470 (12)		6469 (12)	6239 (12)	0.0099	
Pulmonary disease, *n* (%)			<0.001				0.007
No chronic pulmonary disease	264 397 (89)	46 681 (88)		46 679 (88)	46 966 (88)	–0.0135	
Chronic pulmonary disease requiring use of long-term medication	33 722 (11)	6622 (12)		6622 (12)	6335 (12)	0.017	
PVD, *n* (%)			0.76				0.85
No	260 448 (87)	46 542 (87)		46 540 (87)	46 560 (87)	–0.017	
Yes	37 671 (13)	6761 (13)		6761 (13)	6741 (13)	0.0011	
Preop AF, *n* (%)			0.82				0.81
No	288 498 (97)	51 593 (97)		51 591 (97)	51 605 (97)	–0.0011	
Yes	9621 (3.2)	1710 (3.2)		1710 (3.2)	1696 (3.2)	0.0015	
LMS, *n* (%)			0.86				>0.99
No LMS disease or LMS disease ≤50% diameter stenosis	297 753 (100)	53 236 (100)		53 234 (100)	53 ) (100)	–0.0015	
LMS >50% diameter stenosis	366 (0.1)	67 (0.1)		67 (0.1)	67 (0.1)	0	
Neuro Dys, *n* (%)			0.001				0.003
No	292 159 (98)	52 351 (98)		52 349 (98)	52 214 (98)	0	
Yes	5960 (2.0)	952 (1.8)		952 (1.8)	1087 (2.0)	–0.0181	
CrCl. category, *n* (%)			<0.001				<0.001
Severe (CC <50 ml/min)	7462 (2.5)	1242 (2.3)		1242 (2.3)	1328 (2.5)	0.0181	
Moderate (CC 50–85 ml/m)	40 705 (14)	5780 (11)		5780 (11)	6683 (13)	0.0103	
Normal (CC >85 ml/min)	249 068 (84)	46 088 (86)		46 087 (86)	45 115 (85)	0.0493	
CCS class, *n* (%)			<0.001				<0.001
0	30 122 (10)	4825 (9.1)		4825 (9.1)	5235 (9.8)	–0.0059	
1	26 716 (9.0)	5047 (9.5)		5047 (9.5)	4868 (9.1)	0.0255	
2	107 809 (36)	19 088 (36)		19 088 (36)	19 168 (36)	–0.0118	
3	93 891 (31)	17 143 (32)		17 142 (32)	16 767 (31)	0.0031	
4	39 581 (13)	7200 (14)		7199 (14)	7263 (14)	–0.0151	
NYHA class, *n* (%)			<0.001				0.32
1	92 530 (31)	16 812 (32)		16 811 (32)	16 581 (31)	0.0035	
2	137 039 (46)	24 004 (45)		24 004 (45)	24 173 (45)	–0.0093	
3	60 300 (20)	11 105 (21)		11 104 (21)	11 104 (21)	0.0064	
4	8250 (2.8)	1382 (2.6)		1382 (2.6)	1443 (2.7)	0	
PCI, *n* (%)			<0.001				<0.001
No previous PCI	268 845 (90)	47 150 (88)		47 150 (88)	47 627 (89)	0.007	
PCI <24 h before surgery	818 (0.3)	201 (0.4)		200 (0.4)	186 (0.3)	0.0301	
PCI >24 h before surgery; same admission	2 607 (0.9)	530 (1.0)		530 (1.0)	481 (0.9)	–0.005	
PCI >24 h before surgery; previous admission	25 849 (8.7)	5422 (10)		5421 (10)	5007 (9.4)	–0.0099	
Previous MI, *n* (%)			<0.001				<0.001
None	159 311 (53)	29 201 (55)		29 201 (55)	28 454 (53)	–0.0276	
One	115 482 (39)	20 130 (38)		20 129 (38)	20 632 (39)	–0.0281	
Two or more	23 326 (7.8)	3972 (7.5)		3971 (7.5)	4215 (7.9)	0.0194	
Poor mobility, *n* (%)			<0.001				0.003
No	291 727 (98)	52 315 (98)		52 313 (98)	52 176 (98)	0.017	
Yes	6392 (2.1)	988 (1.9)		988 (1.9)	1125 (2.1)	–0.0177	
Interval MI, *n* (%)			<0.001				<0.001
No previous MI	227 209 (76)	41 360 (78)		41 360 (78)	40 769 (76)	0.0177	
MI <6 h	195 (<0.1)	52 (<0.1)		51 (<0.1)	39 (<0.1)	–0.026	
MI 6–24 h	744 (0.2)	171 (0.3)		170 (0.3)	159 (0.3)	–0.0088	
MI 1–30 days	48 410 (16)	7673 (14)		7673 (14)	8358 (16)	–0.0041	
MI 31–90 days	21 561 (7.2)	4047 (7.6)		4047 (7.6)	3976 (7.5)	0.0348	
Ventilated preop, *n* (%)			0.55				>0.99
No	297 730 (100)	53 228 (100)		53 226 (100)	53 226 (100)	–0.0051	
Yes	389 (0.1)	75 (0.1)		75 (0.1)	75 (0.1)	0	
Cardiogenic shock, *n* (%)			0.013				0.65
No	297 270 (100)	53 184 (100)		53 182 (100)	53 175 (100)	0	
Yes	849 (0.3)	119 (0.2)		119 (0.2)	126 (0.2)	–0.0025	
Inotropes, *n* (%)			0.36				0.73
No	296 850 (100)	53 091 (100)		53 089 (100)	53 096 (100)	0.0025	
Yes	1269 (0.4)	212 (0.4)		212 (0.4)	205 (0.4)	0.002	

AF: atrial fibrillation; BMI: body mass index; CCS: Canadian Cardiovascular Society; CrCl.: creatinine clearance; Inotropes: inotropic support prior to general anaesthesia; LMS: left main stem disease; LVEF: left ventricular ejection fraction; MI: myocardial infarction; NYHA: New York Heart Association; Neuro Dys: neurological dysfunction; ONCAB: on-pump coronary artery bypass; OPCAB: off-pump coronary artery bypass graft; PCI: percutaneous coronary intervention; PSM: propensity score matching; PVD: peripheral vascular disease; SD: standard deviation; SMD: standardized mean difference; ventilated preop: require invasive ventilation (including intubation) preoperatively.

## RESULTS

**Figure 1:**
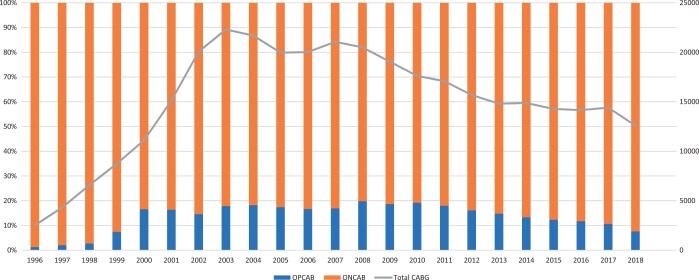
The proportion of off-pump coronary artery bypass graft (blue) and on-pump coronary artery bypass (orange) and total number of isolated coronary artery bypass graft (grey) performed in the UK from 2000 to 2018.

**Figure 2:**
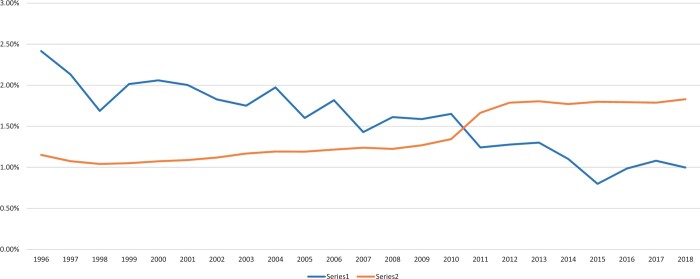
The predicted mortality rate (Series 2) and the observed mortality rate (Series 1) for all isolated coronary artery bypass graft from 2000 to 2018.

**Figure 3:**
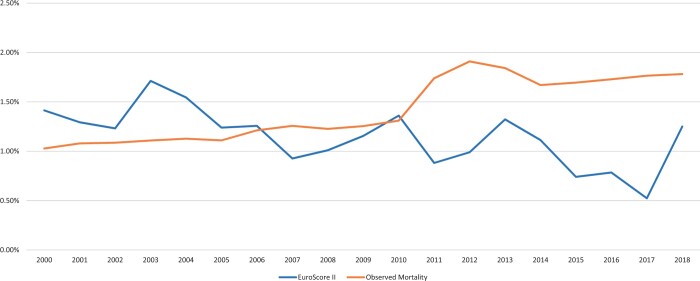
The predicted mortality rate (Series 2) and the observed mortality rate (Series 1) for off-pump coronary artery bypass graft from 2000 to 2018.

A total of 351 422 patients were included in this study, of whom 53 301 (15.17%) underwent OPCAB. The number of OPCAB performed increased in the early 2000s. After it peaked in 2008, to 19.80%, the number gradually decreased afterwards to 7.63% in 2018 (Fig. 1). The mortality rate for all isolated CABG was 1.52% over the study period. The observed mortality rate was reduced from ∼2% in early 2000s to 1.12% in 2018 while the EuroScore II increased from 1.07% in 2000 to 1.82% in 2018. Figures 2 and 3 show, respectively, the observed and predicted mortality in all isolated CABG and separately for OPCAB from 2000 to 2018.

### Perioperative characteristics (1996–2019)

PSM created 2 equally sized groups (*n* = 53 303 in each group) and the preoperative characteristics are shown in Table 1. After PSM, patients who underwent OPCAB were more likely to be female (80% vs 79%, *P* = 0.003), had a lower body mass index [28.33 (SD: 4.68) vs 28.48 (SD: 4.55), *P* < 0.001], performed in an elective setting [68% vs 66%, *p* < 0.001], with preoperative renal impairment (creatinine >200) [2.0% vs 1.8%, *P* = 0.035] and a lower EuroScore II [1.35 (SD: 1.36) vs 1.39 (SD: 1.39), *P* < 0.001]. There were no differences in age [65.81 (SD: 9.94) vs 65.84 (SD: 9.51) years, *P* = 0.67], incidence of preoperative peripheral vascular disease [13% vs 13%, *P* = 0.97] and preoperative atrial fibrillation [3.2% vs 3.2%, *P* = 0.88] between OPCAB and ONCAB.

The mean cardiopulmonary bypass and aortic cross-clamp time in the ONCAB group was 83.81 (SD 33.70 min) and 48.80 (SD 21.76 min), respectively. The median and mean number of distal anastomosis performed in OPCAB and ONCAB were 3 and 2.51 and 3 and 3.01, respectively, *P* < 0.001. OPCAB was associated with a higher rate of multiple arterial grafting [34% vs 8.5%, *P* < 0.001].

OPCAB when compared with ONCAB was associated with a lower in-hospital/30-day mortality [1.2% vs 1.5%, *P* < 0.001], incidence of return to theatre [3.7% vs 4.7%, *P* < 0.001], postoperative CVA [TIA: 0.3% vs 0.5%, stroke: 0.3% vs 0.7%, *P* < 0.001] and deep sternal wound infection [0.8% vs 1.1%, *P* = 0.016]. No differences were seen in the incidence of postoperative dialysis [1.9% vs 2.0%, *P* = 0.18]. Table 2 shows the postoperative characteristics between ONCAB and OPCAB.

**Table 2:** The intra- and postoperative outcome between off-pump coronary artery bypass graft and on-pump coronary artery bypass after propensity score matching (1996–2019)

Intra- and postoperative characteristics	Pre-PSM			Post-PSM		
Variable	ONCAB (*n* = 298 119)	OPCAB (*n* = 53 303)	*P*-Value	ONCAB (*n* = 53 301)	OPCAB (*n* = 53 301)	*P*-Value
CPB, mean (SD)	83.81 (33.70)	N/A	N/A	48.53 (21.49)	N/A	N/A
XClamp, mean (SD)	48.80 (21.76)	N/A	N/A	83.52 (34.14)	N/A	N/A
Number grafts, *n* (%)			<0.001			<0.001
0	3500 (1.2)	885 (1.7)		615 (1.2)	885 (1.7)	
1	8389 (2.8)	8243 (16)		1557 (3.0)	8242 (16)	
2	58 067 (20)	15 725 (30)		10 584 (20)	15 725 (30)	
3	146 578 (50)	20 177 (38)		26 105 (49)	20 176 (38)	
4	68 625 (23)	6539 (12)		12 175 (23)	6539 (12)	
5	9204 (3.1)	1076 (2.0)		1603 (3.0)	1076 (2.0)	
6	779 (0.3)	167 (0.3)		137 (0.3)	167 (0.3)	
Arterial grafts, *n* (%)						<0.001
0	30 434 (10)	2955 (5.5)		5628 (11)	2955 (5.5)	
1	232 217 (78)	34 152 (64)		41 266 (77)	34 150 (64)	
2	31 624 (11)	14 629 (27)		5684 (11)	14 629 (27)	
3	3839 (1.3)	1558 (2.9)		721 (1.4)	1558 (2.9)	
4	5 (<0.1)	9 (<0.1)		2 (<0.1)	9 (<0.1)	
Mortality, *n* (%)	4564 (1.5)	632 (1.2)	<0.001	818 (1.5)	632 (1.2)	<0.001
RTT, *n* (%)	11 977 (4.6)	1844 (3.7)	<0.001	2085 (4.5)	1844 (3.7)	<0.001
RTT, bleeding, tamponade, *n* (%)	8668 (2.9)	1210 (2.3)	<0.001	1484 (2.8)	1210 (2.3)	<0.001
Postop CVA, *n* (%)			<0.001			<0.001
0. None	259 837 (99)	46 153 (99)		46 470 (99)	46 151 (99)	
1. Transient stroke	1474 (0.6)	141 (0.3)		263 (0.6)	141 (0.3)	
2. Permanent stroke	1735 (0.7)	160 (0.3)		300 (0.6)	160 (0.3)	
Postop dialysis, *n* (%)	5096 (2.0)	897 (1.9)	0.49	886 (1.9)	897 (1.9)	0.92
Postop DSWI, *n* (%)	811 (1.1)	80 (0.8)	0.001	158 (1.2)	80 (0.8)	<0.001
Postop DSWI treatment, *n* (%)			0.004			<0.001
None	72 797 (99)	10 453 (99)		12 624 (99)	10 452 (99)	
Conservative Rx (Vac Pump)	321 (0.4)	28 (0.3)		59 (0.5)	28 (0.3)	
Surgical debridement	304 (0.4)	29 (0.3)		68 (0.5)	29 (0.3)	
Total length of stay (days), mean (SD)	11.88 (22.62)	10.71 (10.22)	<0.001	11.87 (11.62)	10.71 (10.22)	<0.001

CPB: cardiopulmonary bypass; CVA: cerebrovascular accident; DSWI: deep sternal wound infection; N/A: not applicable; ONCAB: on-pump coronary artery bypass grafting; OPCAB: off-pump coronary artery bypass grafting; RTT: return to theatre; PSM: propensity score matching; SD: standard deviation; XClamp: cross-clamp.

In a subgroup analysis in patients with preoperative pulmonary disease, extracardiac arteriopathy and EuroScore II ≥4, we found no differences in-hospital/30 day mortality or incidence of deep sternal wound infection between the 2 groups. However, a short-term mortality benefit was shown in patients with poor left ventricular dysfunction (2.3% vs 3.0%, *P* = 0.001) undergoing OPCAB. The incidence of CVA in the OPCAB group was lower across all high-risk groups except for patients with EuroScore II ≥4. Patients with poor LV and pulmonary disease who underwent OPCAB had a lower incidence of return to theatre [4.4% vs 5.8%, *P* < 0.001, and 4.6% vs 5.7%, *P* = 0.007, respectively].

### Off-pump coronary artery bypass in the EuroScore II era (2012–2019)

When comparing ONCAB and OPCAB performed after 2012, there were no differences in lower in-hospital/30-day mortality [1.0% vs 1.0%, *P* = 0.71] and postoperative dialysis [1.7% vs 1.5%, *P* = 0.28]. However, ONCAB had a higher incidence of return to theatre [4.2% vs 2.7%, *P* < 0.001], postoperative CVA [TIA: 0.4% vs 0.2%, stroke: 0.5% vs 0.3%, *P* = 0.003] and deep sternal wound infection [1.0% vs 0.6%, *P* = 0.004] (Table 3). The preoperative characteristics of this cohort are presented in Supplementary Material, Table S1.

**Table 3:** The intra- and postoperative outcome between off-pump coronary artery bypass graft and on-pump coronary artery bypass in cases performed between 2012 and 2019 after propensity score matching

Intra- and postoperative characteristics	Pre-PSM			Post-PSM		
Variable	ONCAB (*n* = 90 941)	OPCAB (*n* = 12 779)	*P*-Value	ONCAB (*n* = 12 776)	OPCAB (*n* = 12 776)	*P*-Value
CPB (min), mean (SD)	88.77 (35.47)	N/A	N/A	54.31 (22.77)	N/A	N/A
XClamp time (min), mean (SD)	54.21 (22.71)	N/A	N/A	88.97 (38.07)	N/A	N/A
Number grafts, *n* (%)			<0.001			<0.001
0	184 (0.2)	39 (0.3)		28 (0.2)	39 (0.3)	
1	1838 (2.0)	1935 (15)		275 (2.2)	1935 (15)	
2	18 753 (21)	3972 (32)		2618 (21)	3971 (32)	
3	45 741 (51)	4577 (37)		6359 (50)	4575 (37)	
4	20 621 (23)	1581 (13)		2921 (23)	1581 (13)	
5	2594 (2.9)	344 (2.7)		382 (3.0)	344 (2.7)	
6	200 (0.2)	74 (0.6)		29 (0.2)	74 (0.6)	
Arterial grafts, *n* (%)						<0.001
0	7878 (8.7)	665 (5.2)		1114 (8.7)	665 (5.2)	
1	76 150 (84)	9244 (72)		10 668 (84)	9242 (72)	
2	6200 (6.8)	2611 (20)		883 (6.9)	2610 (20)	
3	712 (0.8)	259 (2.0)		111 (0.9)	259 (2.0)	
4	1 (<0.1)	0 (0)		1266 (9.9)	4174 (33)	
Mortality, *n* (%)	978 (1.1)	124 (1.0)	0.36	131 (1.0)	123 (1.0)	0.71
Return to theatre, *n* (%)	3332 (4.0)	333 (2.7)	<0.001	490 (4.2)	333 (2.7)	<0.001
Return to theatre due to bleeding, *n* (%)	2552 (2.8)	233 (1.8)	<0.001	381 (3.0)	233 (1.8)	<0.001
Postop CVA, *n* (%)			<0.001			0.003
None	81 643 (99)	10 888 (99)		11 436 (99)	10 885 (99)	
Transient stroke	323 (0.4)	24 (0.2)		43 (0.4)	24 (0.2)	
Permanent stroke	440 (0.5)	31 (0.3)		58 (0.5)	31 (0.3)	
Postop dialysis, *n* (%)	1351 (1.6)	186 (1.7)	0.63	175 (1.5)	185 (1.7)	0.28
Postop DSWI, *n* (%)	655 (1.0)	57 (0.6)	<0.001	94 (1.0)	57 (0.6)	0.004
Postop DSWI, treatment, *n* (%)			<0.001			0.002
None	63 579 (99)	8770 (100)		8948 (99)	8768 (100)	
Conservative Rx (Vac Pump)	276 (0.4)	22 (0.2)		43 (0.5)	22 (0.2)	
Surgical debridement	267 (0.4)	16 (0.2)		35 (0.4)	16 (0.2)	
Total length of stay (days), mean (SD)	11.89 (10.43)	10.78 (9.99)	<0.001	11.90 (10.36)	10.78 (9.99)	<0.001

CPB: cardiopulmonary bypass; CVA: cerebrovascular accident; DSWI: deep sternal wound infection; N/A: not applicable; OPCAB: off-pump coronary artery bypass grafting; ONCAB: on-pump coronary artery bypass grafting; PSM: propensity score matching; RTT: return to theatre; SD: standard deviation; XClamp: cross-clamp.

### Proportion of off-pump coronary artery bypass performed by trainees, individual hospital units and surgeons

Trainee exposure to OPCAB followed a similar pattern to the number of total OPCAB performed. The number of OPCAB performed by trainees peaked in 2009 with nearly 4% and then significantly reduced after 2013, with an average between 1.5% and 2% with trainees being the primary operator. Overall, 2.46% of OPCAB were performed by trainee compared with 26.2% of ONCAB.

**Figure 4:**
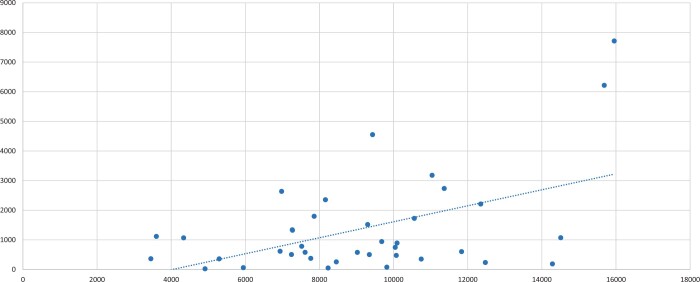
The number of total isolated coronary artery bypass graft recorded (*x*-axis) and the proportion of cases performed using off-pump coronary artery bypass graft (*y*-axis) in each individual centre in the UK.

The proportion of OPCAB performed varied significantly between centres, ranging from 0.52% to 48.36% of total cases. The 2 highest volume centres performed >40% of their cases off pump, while the 3rd and 4th highest performed <10% of cases (Fig. 4). Similarly, the top 2 surgeons, who recorded >2400 cases each, performed ∼80% and 65% of their cases off pump. Of the 82 surgeons who recorded >1000 isolated CABG, 15 (18%) performed more than half of their overall cases using off-pump technique (Table 4). More than half surgeons (*n* = 45) performed >95% of their cases with cardiopulmonary bypass. Table 5 shows the number of OPCAB performed in centres which logged >10000 cases.

**Table 4:** The 15 surgeons with the highest number of isolated coronary artery bypass graft logged and the proportion of cases performed using off-pump coronary artery bypass graft

Surgeon	OPCAB	Total isolated CABG	Proportion (%)
1	1550	2528	61.31
2	1887	2493	75.69
3	587	2373	24.74
4	0	2211	0.00
5	208	2187	9.51
6	401	2137	18.76
7	110	2081	5.29
8	0	1981	0.00
9	1312	1972	66.53
10	1243	1891	65.73
11	79	1883	4.20
12	660	1881	35.09
13	3	1862	0.16
14	1639	1851	88.55
15	3	1806	0.17

CABG: coronary artery bypass graft; OPCAB: off-pump coronary artery bypass graft.

## DISCUSSION

Our study demonstrated a reduction in popularity in OPCAB across the study period in the UK. Several reasons could explain our findings:

First, OPCAB has failed to demonstrate short and long-term survival benefits compared with ONCAB in RCTs. No differences were shown in terms of symptoms, generic and disease-specific quality of life between OPCAB and ONCAB in the midterm follow-up in the BHACAS (Beating heart against cardioplegic arrest studies) 1 and 2 trials [8]. The long-term follow-up in ROBBY and CORONARY trial showed no advantages in terms of survival and repeat revascularization between the 2 techniques [9, 10]. Similar results were reported in the subgroup analysis from the ART trial [11]. Raja *et al.* in a single-centre study reported their 20-year follow-up comparing OPCAB and ONCAB which, again, showed no differences between the 2 techniques [12]. In fact, OPCAB was often associated with a lower number of graft and increased risk of incomplete revascularization. Incomplete revascularization is known to be independently associated with a poorer long-term survival and increased the need for repeat revascularization [13–15]. The analysis for the time period 1996–2019 showed that OPCAB, when compared to ONCAB, was associated with a significantly lower in-hospital/30-day mortality. However, similar to most retrospective studies, and RCTs no differences between ONCAB and OPCAB were identified in early mortality when a sub-analysis was conducted after the introduction of EuroScore II (2012–2019).

Although most studies rightly focuses on mortality, incidence of repeat revascularization and major adverse cardiovascular events (MACEs), there seems to be general agreement that OPCAB reduces the incidence of early clinical outcomes like low cardiac output, postoperative renal dysfunction, need for red cell transfusion and length of intensive care unit and hospital stay [16]. We found that patients undergoing OPCAB had a reduced incidence of return to theatre, deep sternal wound infection and postoperative cerebral vascular accident (CVA). OPCAB also seems to be of benefit in several high-risk groups. Naito *et al.* showed that, in experience OPCAB centres, there was a lower post-procedural acute kidney injury, shorter duration of intensive care unit stay and lower 30-day mortality [17]. In patients with high surgical risk, meta-analyses have observed reduced perioperative morbidity and mortality [18], especially for those in the highest-risk quartiles [19]. Similarly, in large retrospective studies, patients with impaired ventricular function undergoing OPCAB have shown a reduced risk in mortality, CVA and MACEs [20, 21]. As a result, the 2018 ESC/EACTS Guidelines on myocardial revascularization recommended OPCAB for subgroups of high-risk patients to be performed by experienced off-pump teams (class IIa, level B) [22].

The trend in OPCAB varies worldwide. In the USA, OPCAB use has reduced to almost half of the initial rate over a 15-year period in Veterans Affairs medical centres [5]. Only 8/43 (18%) centres used OPCAB for >30% of the total cases and 3/25 (12%) did not perform OPCAB at all. A similar trend was reported by the STS (Society of Thoracic Surgeons) database. Only 17 867 (11.66%) of CABG was performed off pump in 2022, a reduction from 30 730 (19.11%) in 2010. Following a different trend, the use of OPCAB in Germany has increased from 13.1% in 2009 to 23.85% in 2021 [23]. The OPCAB reported rate was 53–61.0% in Korea [24, 25] and 55.0% in Japan [26].

The trend in the UK indicates that OPCAB is performed routinely by a small group of surgeons. Only 18% of surgeons, who logged >1000 procedures, performed >50% of their cases without the use of cardiopulmonary bypass. Similarly, only 2 units performed >40% of the total cases using off-pump technique. Multiple studies have reported that the volume of OPCAB plays an important role in clinical outcomes. Benedetto *et al.* [27] suggested that clinical outcomes of OPCAB are highly dependent on the volume of OPCAB performed at both the institution and the individual surgeon levels. A sub-analysis in the ART trials also suggested a similar 10-year outcome between the 2 revascularization technique when performed by experienced surgeons [11].

OPCAB is not an easy surgery, and it is acknowledged that it involves a great deal of surgical dexterity and skill [28]. Similar to multiple arterial revascularization, OPCAB requires dedication, infrastructure, and expertise to achieve proficiency and good results. This in turn raises the question: can OPCAB simply be introduced into routine clinical practice, or should it be a specialized technique? This, again, raises the question on the need for coronary specialists in each individual centre [4].

Percutaneous coronary interventions are now being routinely offered to patients with multivessel and/or left main coronary artery disease. Therefore, future patients requiring CABG will more likely have complex coronary artery disease [17, 22]. Furthermore, it is expected that the incidence of high-risk profile patients with atherosclerotic aorta, reduced left ventricular ejection fraction, stroke and/or renal failure will continue to rise. Having a unit experienced OPCAB team that performs OPCAB routinely would benefit this cohort of patients.

A lack of exposure to OPCAB in the training program is likely to be another reason to explain the observed trend. Previous studies showed that OPCAB can be safely performed by trainee under supervision by senior surgeons within a dedicated training program [29, 30]. However, except for a few dedicated units that offer fellowship training, to the best of our knowledge, there are no national societies that have included OPCAB in the training curriculum [3, 4]. An unpublished trainee survey in the UK (75% response rate, *n* = 105) showed that training in OPCAB is variable across training programs with only 23.1% of respondents receiving formal training. This correlates with our finding of <2% of OPCAB performed by trainees in the past few years. Without a dedicated OPCAB training fellowship by coronary specialists, this decreasing trend is likely to continue.

### Limitations

There are several limitations to our study. This study is subject to all the limitations associated with observation medical study with operations extending over 23 years. During this time, the perioperative management and surgical techniques for both OPCAB and ONCAB have evolved significantly. The anatomy of the coronaries was not recorded on the database and this could have influenced the decision to perform the operation with/without the use of cardiopulmonary bypass. Another major limitation is the conversion from off pump to on pump which is known to increase the risk of death and serious complications. However, from our experience, the conversion is around <1%, and given the large number of patients included in the analysis, the effect should also be minimal [31].

The NACSA database heavily relies on healthcare professionals’ input and some data were missing in some parts of the analysis. This is particularly apparent in the postoperative outcome and some of the non-mandatory options in the database. A small amount (<5%) of data on cardiopulmonary bypass use was missing and required to be excluded from the analysis. Despite the application of PSM, residual bias may be present in the analysis since the propensity-matched model can account only for measured confounders and not for the unmeasured confounders (e.g. frailty). The absence of conversion from off pump to on pump, long-term follow-up data showing survival rate, the need for revascularization and major adverse cardiac events rate are another limitation. Nevertheless, we believe that our study is an important topic in surgical practice worldwide.

## CONCLUSION

Our data show a decreasing trend in the use of OPCAB in the UK since 2008. This reduction is likely to be multifactorial and raises the question of whether OPCAB should be a specialized revascularization technique. OPCAB remains a good complementary coronary revascularization technique with low mortality and early clinical complications.

## Supplementary Material

ezad272_Supplementary_Data

## Data Availability

The data underlying this article were provided by National Institute for Cardiovascular Outcomes Research by permission. Data will be shared on request to the corresponding author with the permission of National Institute for Cardiovascular Outcomes Research.

## ABBREVIATIONS

CABG: Coronary artery bypass grafting
CI: Confidence interval
NACSA: National Adult Cardiac Surgery Audit
ONCAB: On-pump coronary artery bypass
OPCAB: Off-pump coronary artery bypass
OR: Odds ratio
PSM: Propensity score matching
RCTs: Randomized control trials
SD: Standard deviation

## References

[1] Goetz RH , Rohman M, Haller JD, Dee R, Rosenak SS. Internal mammary-coronary artery anastomosis. A nonsuture method employing tantalum rings. J Thorac Cardiovasc Surg 1961;41:378–86. 13706288

[2] Fudulu D , Benedetto U, Pecchinenda GG, Chivasso P, Bruno VD, Rapetto F et al Current outcomes of off-pump versus on-pump coronary artery bypass grafting: evidence from randomized controlled trials. J Thorac Dis 2016;8:S758–S771. 27942394 10.21037/jtd.2016.10.80 PMC5124584

[3] Angelini GD. An old off-pump coronary artery bypass surgeon's reflections: a retrospective. J Thorac Cardiovasc Surg 2019;157:2274–7. 30396736 10.1016/j.jtcvs.2018.09.086

[4] Farina P , Gaudino M, Angelini GD. Off-pump coronary artery bypass surgery: the long and winding road. Int J Cardiol 2019;279:51–5. 30318295 10.1016/j.ijcard.2018.09.101

[5] Deo SV , Elgudin Y, Shroyer ALW, Altarabsheh S, Sharma V, Rubelowsky J et al Off-pump coronary artery bypass grafting: department of veteran affairs' use and outcomes. J Am Heart Assoc 2022;11:e023514. 35229663 10.1161/JAHA.121.023514 PMC9075317

[6] Bakaeen FG , Shroyer AL, Gammie JS, Sabik JF, Cornwell LD, Coselli JS et al Trends in use of off-pump coronary artery bypass grafting: results from the Society of Thoracic Surgeons Adult Cardiac Surgery Database. J Thorac Cardiovasc Surg 2014;148:856. 25043865 10.1016/j.jtcvs.2013.12.047

[7] Benedetto U , Sinha S, Dimagli A, Cooper G, Mariscalco G, Uppal R et al; UK Aortic Group. Decade-long trends in surgery for acute Type A aortic dissection in England: a retrospective cohort study. Lancet Reg Health Eur 2021;7:100131. 34557840 10.1016/j.lanepe.2021.100131 PMC8454541

[8] Ascione R , Reeves BC, Taylor FC, Seehra HK, Angelini GD. Beating heart against cardioplegic arrest studies (BHACAS 1 and 2): quality of life at mid-term follow-up in two randomised controlled trials. Eur Heart J 2004;25:765–70. 15120887 10.1016/j.ehj.2003.11.015

[9] Quin JA , Wagner TH, Hattler B, Carr BM, Collins J, Almassi GH et al Ten-year outcomes of off-pump vs on-pump coronary artery bypass grafting in the Department of Veterans Affairs: a randomized clinical trial. JAMA Surg 2022;157:303–10. 35171210 10.1001/jamasurg.2021.7578 PMC8851363

[10] Lamy A , Devereaux PJ, Prabhakaran D, Taggart DP, Hu S, Straka Z et al; CORONARY Investigators. Five-year outcomes after off-pump or on-pump coronary-artery bypass grafting. N Engl J Med 2016;375:2359–68. 27771985 10.1056/NEJMoa1601564

[11] Taggart DP , Gaudino MF, Gerry S, Gray A, Lees B, Sajja LR et al; Arterial Revascularization Trial Investigators. Ten-year outcomes after off-pump versus on-pump coronary artery bypass grafting: insights from the Arterial Revascularization Trial. J Thorac Cardiovasc Surg 2021;162:591–9 e8. 32173100 10.1016/j.jtcvs.2020.02.035

[12] Raja SG , Garg S, Soni MK, Rochon M, Marczin N, Bhudia SK et al On-pump and off-pump coronary artery bypass grafting for patients needing at least two grafts: comparative outcomes at 20 years. Eur J Cardiothorac Surg 2020;57:512–9. 31549144 10.1093/ejcts/ezz261

[13] Yi G , Youn YN, Joo HC, Hong S, Yoo KJ. Association of incomplete revascularization with long-term survival after off-pump coronary artery bypass grafting. J Surg Res 2013;185:166–73. 23769631 10.1016/j.jss.2013.05.042

[14] Chikwe J , Lee T, Itagaki S, Adams DH, Egorova NN. Long-term outcomes after off-pump versus on-pump coronary artery bypass grafting by experienced surgeons. J Am Coll Cardiol 2018;72:1478–86. 30236310 10.1016/j.jacc.2018.07.029

[15] Angelini GD , Taylor FC, Reeves BC, Ascione R. Early and midterm outcome after off-pump and on-pump surgery in Beating Heart Against Cardioplegic Arrest Studies (BHACAS 1 and 2): a pooled analysis of two randomised controlled trials. Lancet 2002;359:1194–9. 11955537 10.1016/S0140-6736(02)08216-8

[16] Deppe AC , Arbash W, Kuhn EW, Slottosch I, Scherner M, Liakopoulos OJ et al Current evidence of coronary artery bypass grafting off-pump versus on-pump: a systematic review with meta-analysis of over 16,900 patients investigated in randomized controlled trialsdagger. Eur J Cardiothorac Surg 2016;49:1031–41; discussion 1041. 26276839 10.1093/ejcts/ezv268

[17] Naito S , Demal TJ, Sill B, Reichenspurner H, Onorati F, Gatti G et al Impact of surgeon experience and centre volume on outcome after off-pump coronary artery bypass surgery: results from the European Multicenter Study on Coronary Artery Bypass Grafting (E-CABG) Registry. Heart Lung Circ 2022. 10.1016/j.hlc.2022.11.009 36566143

[18] Kowalewski M , Pawliszak W, Malvindi PG, Bokszanski MP, Perlinski D, Raffa GM et al Off-pump coronary artery bypass grafting improves short-term outcomes in high-risk patients compared with on-pump coronary artery bypass grafting: meta-analysis. J Thorac Cardiovasc Surg 2016;151:60–77.e1. 26433633 10.1016/j.jtcvs.2015.08.042

[19] Puskas JD , Thourani VH, Kilgo P, Cooper W, Vassiliades T, Vega JD et al Off-pump coronary artery bypass disproportionately benefits high-risk patients. Ann Thorac Surg 2009;88:1142–7. 19766798 10.1016/j.athoracsur.2009.04.135

[20] Keeling WB , Williams ML, Slaughter MS, Zhao Y, Puskas JD. Off-pump and on-pump coronary revascularization in patients with low ejection fraction: a report from the society of thoracic surgeons national database. Ann Thorac Surg 2013;96:83–8; discussion 88–9. 23743061 10.1016/j.athoracsur.2013.03.098

[21] Ueki C , Miyata H, Motomura N, Sakaguchi G, Akimoto T, Takamoto S. Off-pump versus on-pump coronary artery bypass grafting in patients with left ventricular dysfunction. J Thorac Cardiovasc Surg 2016;151:1092–8. 26725715 10.1016/j.jtcvs.2015.11.023

[22] Neumann FJ , Sousa-Uva M, Ahlsson A, Alfonso F, Banning AP, Benedetto U et al; ESC Scientific Document Group. 2018 ESC/EACTS Guidelines on myocardial revascularization. Eur Heart J 2019;40:87–165.30165437

[23] Beckmann A , Meyer R, Lewandowski J, Markewitz A, Blaßfeld D, Böning A. German Heart Surgery Report 2021: the annual updated registry of the German Society for Thoracic and Cardiovascular Surgery. Thorac Cardiovasc Surg 2022;70:362–76. 35948014 10.1055/s-0042-1754353

[24] Park SJ , Jo AJ, Kim HJ, Cho S, Ko MJ, Yun SC et al Real-world outcomes of on- vs off-pump coronary bypass surgery: result from Korean Nationwide Cohort. Ann Thorac Surg 2022;113:1989–98. 34400133 10.1016/j.athoracsur.2021.07.035

[25] Kim HJ , Chung JE, Jung JS, Kim IS, Son HS. Current status of off-pump coronary artery bypass grafting in patients with multiple coronary artery disease compared with on-pump coronary artery bypass grafting: the Korean National Cohort Study. Thorac Cardiovasc Surg 2018;66:470–6. 29852507 10.1055/s-0038-1651516

[26] Saito A , Hirahara N, Motomura N, Miyata H, Takamoto S. Current Status of cardiovascular surgery in Japan, 2015 and 2016: a report based on the Japan Cardiovascular Surgery Database. 2-Isolated coronary artery bypass grafting surgery. Gen Thorac Cardiovasc Surg 2019;67:736–41. 31256329 10.1007/s11748-019-01162-y

[27] Benedetto U , Lau C, Caputo M, Kim L, Feldman DN, Ohmes LB et al Comparison of outcomes for off-pump versus on-pump coronary artery bypass grafting in low-volume and high-volume centers and by low-volume and high-volume surgeons. Am J Cardiol 2018;121:552–7. 29291888 10.1016/j.amjcard.2017.11.035

[28] Tempe DK , Gandhi DA. Time for judicious application of off-pump CABG. J Cardiothorac Vasc Anesth 2023;37:6–7. 36319563 10.1053/j.jvca.2022.10.004

[29] Murzi M , Caputo M, Aresu G, Duggan S, Angelini GD. Training residents in off-pump coronary artery bypass surgery: a 14-year experience. J Thorac Cardiovasc Surg 2012;143:1247–53.e1. 22050988 10.1016/j.jtcvs.2011.09.049

[30] Asimakopoulos G , Karagounis AP, Valencia O, Rose D, Niranjan G, Chandrasekaran V. How safe is it to train residents to perform off-pump coronary artery bypass surgery? Ann Thorac Surg 2006;81:568–72. 16427853 10.1016/j.athoracsur.2005.07.054

[31] Reeves BC , Ascione R, Caputo M, Angelini GD. Morbidity and mortality following acute conversion from off-pump to on-pump coronary surgery. Eur J Cardiothorac Surg 2006;29:941–7. 16675245 10.1016/j.ejcts.2006.03.018

