# Supplementary material for: Trend and early clinical outcomes of off-pump coronary artery bypass grafting in the UK
Source: Eur J Cardiothorac Surg. 2023 Jul 31;64(2):ezad272. doi: 10.1093/ejcts/ezad272 (PMC10876163; doi:10.1093/ejcts/ezad272)
Supplement: ezad272_Supplementary_Data [file ezad272_supplementary_data.docx]

Supplementary Materials: Trend and early clinical outcomes of Off Pump Coronary Artery Bypass Grafting in the United Kingdom

Figure S1 shows the covariate balance before and after propensity score matching.


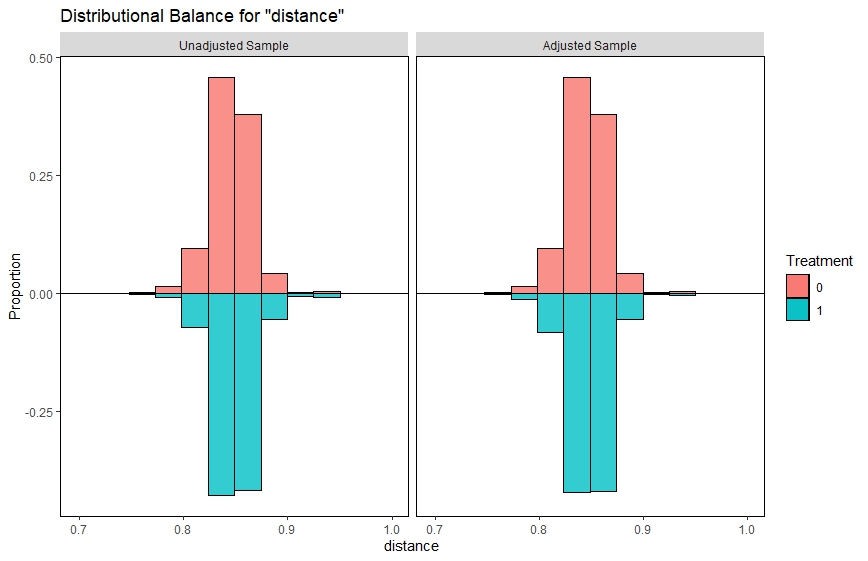


Figure S2 shows the love plot summarising the covariate balance before and after propensity score matching (0: Off pump Coronary artery bypass grafting, 1: On Pump Coronary artery bypass grafting).


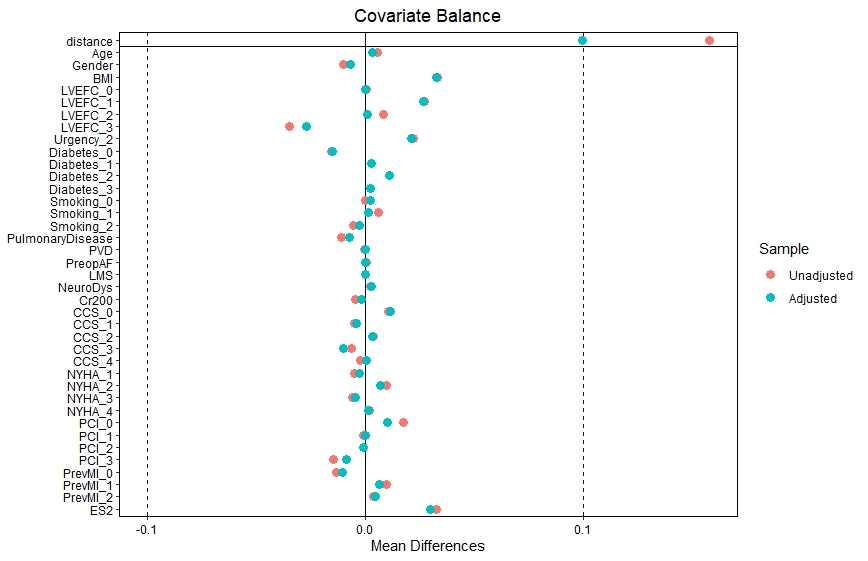


| Pre-operative characteristics | Pre-PSM | | | Post-PSM | | | |
| --- | --- | --- | --- | --- | --- | --- | --- |
|  | ONCAB, (n = 90,941) | OPCAB (N = 12,779) | p-value | ONCAB, (n = 12,776) | OPCAB (n = 12,776) | SMD | p-value |
| Age (years) | 66.60 (9.67) | 66.46 (9.99) | 0.33 | 66.68 (9.82) | 66.46 (9.99) | 0.0149 | 0.11 |
| Gender |  |  | 0.008 |  |  |  | 0.01 |
| Male | 75,145 / 90,941 (83%) | 10,437 / 12,779 (82%) |  | 10,592 / 12,776 (83%) | 10,434 / 12,776 (82%) | 0.0253 |  |
| Female | 15,796 / 90,941 (17%) | 2,342 / 12,779 (18%) |  | 2,184 / 12,776 (17%) | 2,342 / 12,776 (18%) | -0.0253 |  |
|  | 28.94 (5.07) | 28.67 (5.09) | <0.001 | 28.88 (5.00) | 28.67 (5.09) | 0.052 | <0.001 |
| LVEFC |  |  | <0.001 |  |  |  | 0.079 |
| Very Poor (LVEF <21%) | 108 / 90,941 (0.1%) | 3 / 12,779 (<0.1%) |  | 4 / 12,776 (<0.1%) | 3 / 12,776 (<0.1%) | 0.0277 |  |
| Poor (LVEF 21 - 30%) | 19,926 / 90,941 (22%) | 2,813 / 12,779 (22%) |  | 2,754 / 12,776 (22%) | 2,811 / 12,776 (22%) | -0.0025 |  |
| Moderate (LVEF 31 - 50%) | 4,246 / 90,941 (4.7%) | 335 / 12,779 (2.6%) |  | 401 / 12,776 (3.1%) | 335 / 12,776 (2.6%) | 0.097 |  |
| Good (LVEF > 50%) | 66,661 / 90,941 (73%) | 9,628 / 12,779 (75%) |  | 9,617 / 12,776 (75%) | 9,627 / 12,776 (75%) | -0.0461 |  |
| Urgency |  |  | <0.001 |  |  |  | 0.19 |
| Elective | 50,498 / 90,941 (56%) | 7,362 / 12,779 (58%) |  | 7,256 / 12,776 (57%) | 7,360 / 12,776 (58%) | -0.0419 |  |
| Urgent | 40,443 / 90,941 (44%) | 5,417 / 12,779 (42%) |  | 5,520 / 12,776 (43%) | 5,416 / 12,776 (42%) | 0.0419 |  |
| Diabetes |  |  | <0.001 |  |  |  | 0.003 |
| Not Diabetic | 61,873 / 90,941 (68%) | 9,090 / 12,779 (71%) |  | 8,850 / 12,776 (69%) | 9,088 / 12,776 (71%) | -0.0664 |  |
| Diet Control | 4,011 / 90,941 (4.4%) | 513 / 12,779 (4.0%) |  | 580 / 12,776 (4.5%) | 513 / 12,776 (4.0%) | 0.0193 |  |
| Oral therapy | 17,404 / 90,941 (19%) | 2,155 / 12,779 (17%) |  | 2,328 / 12,776 (18%) | 2,154 / 12,776 (17%) | 0.0578 |  |
| Insulin therapy | 7,653 / 90,941 (8.4%) | 1,021 / 12,779 (8.0%) |  | 1,018 / 12,776 (8.0%) | 1,021 / 12,776 (8.0%) | 0.0153 |  |
| Smoking |  |  | 0.35 |  |  |  | 0.73 |
| Never smoked | 32,886 / 90,941 (36%) | 4,703 / 12,779 (37%) |  | 4,664 / 12,776 (37%) | 4,700 / 12,776 (37%) | -0.0133 |  |
| Ex smoker | 46,414 / 90,941 (51%) | 6,468 / 12,779 (51%) |  | 6,529 / 12,776 (51%) | 6,468 / 12,776 (51%) | 0.0085 |  |
| Current smoker | 11,641 / 90,941 (13%) | 1,608 / 12,779 (13%) |  | 1,583 / 12,776 (12%) | 1,608 / 12,776 (13%) | 0.0065 |  |
| Pulmonary Disease |  |  | 0.004 |  |  |  | 0.35 |
| No chronic pulmonary disease | 80,660 / 90,941 (89%) | 11,223 / 12,779 (88%) |  | 11,270 / 12,776 (88%) | 11,221 / 12,776 (88%) | 0.0275 |  |
| Chronic pulmonary disease requiring use of long-term medication | 10,281 / 90,941 (11%) | 1,556 / 12,779 (12%) |  | 1,506 / 12,776 (12%) | 1,555 / 12,776 (12%) | -0.0275 |  |
| PVD |  |  | <0.001 |  |  |  | 0.005 |
| No | 80,335 / 90,941 (88%) | 11,133 / 12,779 (87%) |  | 11,281 / 12,776 (88%) | 11,132 / 12,776 (87%) | 0.0379 |  |
| Yes | 10,606 / 90,941 (12%) | 1,646 / 12,779 (13%) |  | 1,495 / 12,776 (12%) | 1,644 / 12,776 (13%) | -0.0379 |  |
| PreopAF |  |  | 0.9 |  |  |  | 0.78 |
| No | 88,071 / 90,941 (97%) | 12,373 / 12,779 (97%) |  | 12,362 / 12,776 (97%) | 12,370 / 12,776 (97%) | 0.0012 |  |
| Yes | 2,870 / 90,941 (3.2%) | 406 / 12,779 (3.2%) |  | 414 / 12,776 (3.2%) | 406 / 12,776 (3.2%) | -0.0012 |  |
| LMS |  |  | 0.3 |  |  |  | 0.59 |
| No LMS disease or LMS disease <= 50% diameter stenosis | 90,913 / 90,941 (100%) | 12,773 / 12,779 (100%) |  | 12,768 / 12,776 (100%) | 12,770 / 12,776 (100%) | 0.0092 |  |
| LMS >50% diameter stenosis. | 28 / 90,941 (<0.1%) | 6 / 12,779 (<0.1%) |  | 8 / 12,776 (<0.1%) | 6 / 12,776 (<0.1%) | -0.0092 |  |
| Neuro Dys |  |  | 0.31 |  |  |  | 0.28 |
| No | 88,626 / 90,941 (97%) | 12,473 / 12,779 (98%) |  | 12,443 / 12,776 (97%) | 12,470 / 12,776 (98%) | -0.0096 |  |
| Yes | 2,315 / 90,941 (2.5%) | 306 / 12,779 (2.4%) |  | 333 / 12,776 (2.6%) | 306 / 12,776 (2.4%) | 0.0096 |  |
| CrCl. Category |  |  | <0.001 |  |  |  | 0.06 |
| severe (CC < 50 ml/min) | 6,432 / 90,941 (7.1%) | 998 / 12,779 (7.8%) |  | 940 / 12,776 (7.4%) | 998 / 12,776 (7.8%) | -0.0287 |  |
| moderate (CC 50-85 ml/m) | 35,358 / 90,941 (39%) | 4,718 / 12,779 (37%) |  | 4,917 / 12,776 (38%) | 4,718 / 12,776 (37%) | 0.0402 |  |
| normal ( CC > 85 ml/min) | 48,373 / 90,941 (53%) | 6,910 / 12,779 (54%) |  | 6,776 / 12,776 (53%) | 6,908 / 12,776 (54%) | -0.0177 |  |
| CCS Class |  |  | 0.064 |  |  |  |  |
| 0 | 9,078 / 90,941 (10.0%) | 1,286 / 12,779 (10%) |  | 1,322 / 12,776 (10%) | 1,286 / 12,776 (10%) | -0.0027 | 0.76 |
| 1 | 7,826 / 90,941 (8.6%) | 1,075 / 12,779 (8.4%) |  | 1,101 / 12,776 (8.6%) | 1,075 / 12,776 (8.4%) | 0.0069 |  |
| 2 | 35,219 / 90,941 (39%) | 4,800 / 12,779 (38%) |  | 4,832 / 12,776 (38%) | 4,800 / 12,776 (38%) | 0.0239 |  |
| 3 | 27,299 / 90,941 (30%) | 3,939 / 12,779 (31%) |  | 3,891 / 12,776 (30%) | 3,937 / 12,776 (31%) | -0.0176 |  |
| 4 | 11,519 / 90,941 (13%) | 1,679 / 12,779 (13%) |  | 1,630 / 12,776 (13%) | 1,678 / 12,776 (13%) | -0.0142 |  |
| NYHA Class |  |  | <0.001 |  |  |  |  |
| 1 | 25,153 / 90,941 (28%) | 3,973 / 12,779 (31%) |  | 3,733 / 12,776 (29%) | 3,972 / 12,776 (31%) | -0.0767 | 0.002 |
| 2 | 45,748 / 90,941 (50%) | 5,985 / 12,779 (47%) |  | 6,282 / 12,776 (49%) | 5,985 / 12,776 (47%) | 0.0694 |  |
| 3 | 17,774 / 90,941 (20%) | 2,522 / 12,779 (20%) |  | 2,473 / 12,776 (19%) | 2,520 / 12,776 (20%) | -0.0048 |  |
| 4 | 2,266 / 90,941 (2.5%) | 299 / 12,779 (2.3%) |  | 288 / 12,776 (2.3%) | 299 / 12,776 (2.3%) | 0.0097 |  |
| PCI |  |  | <0.001 |  |  |  |  |
| No previous PCI | 76,519 / 90,941 (84%) | 10,569 / 12,779 (83%) |  | 10,698 / 12,776 (84%) | 10,569 / 12,776 (83%) | 0.0393 | 0.1 |
| PCI < 24 hours before surgery | 258 / 90,941 (0.3%) | 75 / 12,779 (0.6%) |  | 75 / 12,776 (0.6%) | 72 / 12,776 (0.6%) | -0.057 |  |
| PCI > 24 hours before surgery; same admission | 1,283 / 90,941 (1.4%) | 205 / 12,779 (1.6%) |  | 172 / 12,776 (1.3%) | 205 / 12,776 (1.6%) | -0.0164 |  |
| PCI > 24 hours before surgery; previous admission | 12,881 / 90,941 (14%) | 1,930 / 12,779 (15%) |  | 1,831 / 12,776 (14%) | 1,930 / 12,776 (15%) | -0.0269 |  |
| Prev MI |  |  | 0.1 |  |  |  |  |
| None | 44,640 / 90,941 (49%) | 6,329 / 12,779 (50%) |  | 6,285 / 12,776 (49%) | 6,327 / 12,776 (50%) | -0.0088 | 0.17 |
| One | 39,696 / 90,941 (44%) | 5,471 / 12,779 (43%) |  | 5,580 / 12,776 (44%) | 5,471 / 12,776 (43%) | 0.0169 |  |
| Two or more | 6,605 / 90,941 (7.3%) | 979 / 12,779 (7.7%) |  | 911 / 12,776 (7.1%) | 978 / 12,776 (7.7%) | -0.0153 |  |
| Poor Mobility |  |  | 0.032 |  |  |  |  |
| No | 88,194 / 90,941 (97%) | 12,437 / 12,779 (97%) |  | 12,406 / 12,776 (97%) | 12,434 / 12,776 (97%) | -0.0201 | 0.29 |
| Yes | 2,747 / 90,941 (3.0%) | 342 / 12,779 (2.7%) |  | 370 / 12,776 (2.9%) | 342 / 12,776 (2.7%) | 0.0201 |  |
| Interval MI |  |  | 0.002 |  |  |  |  |
| No previous MI | 60,376 / 90,941 (66%) | 8,692 / 12,779 (68%) |  | 8,606 / 12,776 (67%) | 8,689 / 12,776 (68%) | -0.0345 | 0.75 |
| MI < 6 hours | 58 / 90,941 (<0.1%) | 9 / 12,779 (<0.1%) |  | 11 / 12,776 (<0.1%) | 9 / 12,776 (<0.1%) | -0.0026 |  |
| MI 6-24 hours | 311 / 90,941 (0.3%) | 46 / 12,779 (0.4%) |  | 49 / 12,776 (0.4%) | 46 / 12,776 (0.4%) | -0.0031 |  |
| MI 1-30 days | 25,694 / 90,941 (28%) | 3,384 / 12,779 (26%) |  | 3,473 / 12,776 (27%) | 3,384 / 12,776 (26%) | 0.0394 |  |
| MI 31-90 days | 4,502 / 90,941 (5.0%) | 648 / 12,779 (5.1%) |  | 637 / 12,776 (5.0%) | 648 / 12,776 (5.1%) | -0.0055 |  |
| Ventilated Preop |  |  | 0.84 |  |  |  |  |
| No | 90,828 / 90,941 (99.9%) | 12,764 / 12,779 (99.9%) |  | 12,758 / 12,776 (100%) | 12,761 / 12,776 (100%) | -0.002 | 0.6 |
| Yes | 113 / 90,941 (0.1%) | 15 / 12,779 (0.1%) |  | 18 / 12,776 (0.1%) | 15 / 12,776 (0.1%) | 0.002 |  |
| Cardiogenic Shock |  |  | 0.14 |  |  |  |  |
| No | 90,617 / 90,941 (99.6%) | 12,744 / 12,779 (99.7%) |  | 12,740 / 12,776 (100%) | 12,741 / 12,776 (100%) | -0.0138 | 0.91 |
| Yes | 324 / 90,941 (0.4%) | 35 / 12,779 (0.3%) |  | 36 / 12,776 (0.3%) | 35 / 12,776 (0.3%) | 0.0138 |  |
| Inotropes |  |  | 0.5 |  |  |  |  |
| No | 90,707 / 90,941 (99.7%) | 12,742 / 12,779 (99.7%) |  | 12,736 / 12,776 (100%) | 12,739 / 12,776 (100%) | 0.0064 | 0.73 |
| Yes | 234 / 90,941 (0.3%) | 37 / 12,779 (0.3%) |  | 40 / 12,776 (0.3%) | 37 / 12,776 (0.3%) | -0.0064 |  |

Supp Table 1 shows the pre-operative characteristics between Off pump coronary artery bypass graft (OPCAB) and On pump coronary artery bypass (ONCAB) in cases performed after 2012, before and after propensity score matching (AF: Atrial fibrillation, CCS: Canadian Cardiovascular Society, PCI: Percutaneous Coronary Intervention, BMI: Body Mass Index, NYHA: New York Heart Association, LMS: Left Main stem disease, MI: Myocardial infraction, LVEF: Left ventricular ejection fraction, NeuroDys: Neurological Dysfunction, ES2: Euro Score II, PSM: Propensity score matching, SMD: Standardised mean difference, PVD: Peripheral vascular disease, CrCl. category: Creatinine clearance category, Ventilated Pre op: Require invasive ventilation (including intubation) pre operatively, Inotropes: Inotropic support prior to general anaesthesia.
